# The local anesthetic ropivacaine suppresses progression of breast cancer by regulating miR-27b-3p/YAP axis

**DOI:** 10.18632/aging.203160

**Published:** 2021-06-14

**Authors:** Lu Zhao, Shuang Han, Junde Hou, Wenhui Shi, Yonglei Zhao, Yongxue Chen

**Affiliations:** 1 The Department of Anesthesiology, Handan Central Hospital, Handan, China; 2 The Department of Anesthesiology, Hebei General Hospital, Shijiazhuang, China

**Keywords:** breast cancer, progression, ropivacaine, miR-27b-3p, YAP

## Abstract

Breast cancer is a prevalent malignancy with high mortality and poor prognosis. Ropivacaine is a widely used local anesthetic and presents potential anti-tumor activity. Nevertheless, the function of ropivacaine in breast cancer development remains elusive. Here, we tried to investigate the impact of ropivacaine on breast cancer progression and the underlying mechanism. Significantly, we revealed that ropivacaine was able to reduce the proliferation and induce the apoptosis of breast cancer cells. Ropivacaine could attenuate the invasion and migration in the cells. Mechanically, ropivacaine could enhance the miR-27b-3p expression and miR-27b-3p inhibited breast cancer progression in breast cancer cells. MiR-27b-3p targeted YAP in the breast cancer cells. Ropivacaine decreased the breast cancer progression by modulating miR-27b-3p/YAP axis *in vitro*. Ropivacaine could inhibit tumor growth *in vivo*. In conclusion, we discovered that the local anesthetic ropivacaine inhibits the progression of breast cancer *via* the miR-27b-3p/YAP axis. Our finding presents novel insights into the mechanism of ropivacaine inhibiting the development of breast cancer. Ropivacaine may potentially serve as an anti-tumor candidate in the therapeutic strategy of breast cancer.

## INTRODUCTION

Breast cancer is the most prevailing tumor for females and is the second principal reason for tumor-associated mortality globally, accounting for nearly 22% of total tumor incidents and 13.7% of tumor-related deaths of women globally [1, 2]. The occurrence of breast cancer has expanded in numerous developing countries in recent decades, and breast cancer-related metastasis is high, which is the leading reason for the recurrence and mortality of breast cancer cases [3, 4]. The 5-year survival rate is decreased from 85% in cases with early-stage breast cancer to 23% in cases with late-stage breast cancer [5]. Accordingly, although there have been advanced in therapy, prognosis of breast cancer is still poor [6, 7]. Therefore, exploration of more practical anti-breast cancer candidates will help the selection and development of effective therapeutic strategies for breast cancer.

Ropivacaine serves as monohydrate and its anhydride, which is a distinct high-acting amide local anesthetic with levorotatory structure and has been broadly applied in postoperative analgesia, anesthesia, and other areas [8]. Recently, ropivacaine has been reported to repress cancer progression. For instance, ropivacaine remarkably inhibits the GTPases viability in esophageal cancer cells [9]. Ropivacaine is able to increase apoptosis and restrain the proliferation in liver cancer cells [10]. In addition, ropivacaine has shown an anti-proliferative impact on colon cancer cells [11]. But the function of ropivacaine in breast cancer is still elusive.

MicroRNAs (miRNAs) are characterized as small non-coding RNAs with nucleotides of 20 to 25, and remarkably affect various cellular processes [12]. MiRNAs can modulate expression through targeting 3′ untranslated region (3′ UTR) [13, 14]. It has revealed that miRNAs are able to participate in breast cancer [15, 16]. In addition, the previous studies have shown that miR-27b-3p presents a crucial function in suppressing breast cancer [17]. Furthermore, Yes-associated protein (YAP), as a transcriptional co-activator that is abnormally expressed in diverse cancer models, has been implicated as a well-recognized oncogene [18, 19]. Multiple investigations have identified that YAP contributes to the progression of breast cancer [20, 21]. However, correlation of miR-27b-3p/YAP with ropivacaine in the modulation of breast cancer is obscure.

Here, we investigated the molecular mechanisms of ropivacaine inhibiting breast cancer development. We found an innovative anti-breast cancer function of ropivacaine *via* miR-27b-3p/YAP.

## RESULTS

### Ropivacaine suppresses proliferation and stimulates apoptosis of breast cancer cells

**Figure 1:**
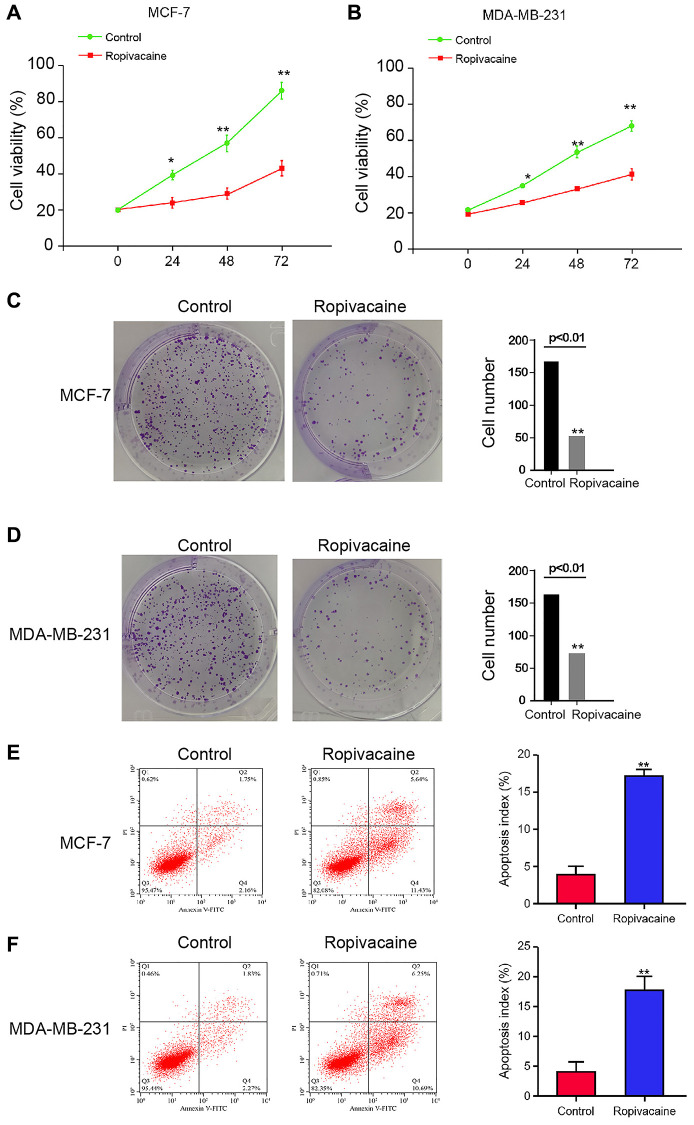
**Ropivacaine inhibits proliferation and promotes apoptosis of breast cancer cells.** (**A**–**F**) The MCF-7 and MDA-MB-231 cells were treated with ropivacaine (1 mmol/L) or equal volume saline. (**A** and **B**) The cell viability was analyzed by the MTT assays in the cells. (**C** and **D**) The cell proliferation was measured by the colony formation assays in the cells. (**E** and **F**) The cell apoptosis was measure by flow cytometry analysis in the cells. *N* = 3, The independent experiments were repeated for three times. Data are presented as mean ± SD. Statistic significant differences were indicated: ^*^P < 0.05, ^**^P < 0.01.

To determine role of ropivacaine in modulating breast cancer, MDA-MB-231 and MCF-7 cells were administrated with ropivacaine. Treatment of ropivacaine repressed cell viabilities (Figure 1A and 1B). The colony numbers were suppressed by the ropivacaine (Figure 1C and 1D). Cell apoptosis was promoted by ropivacaine in the cells (Figure 1E and 1F). It suggests that ropivacaine inhibited proliferation and induces apoptosis of breast cancer cells.

### Ropivacaine decreases breast cancer invasion and migration

**Figure 2:**
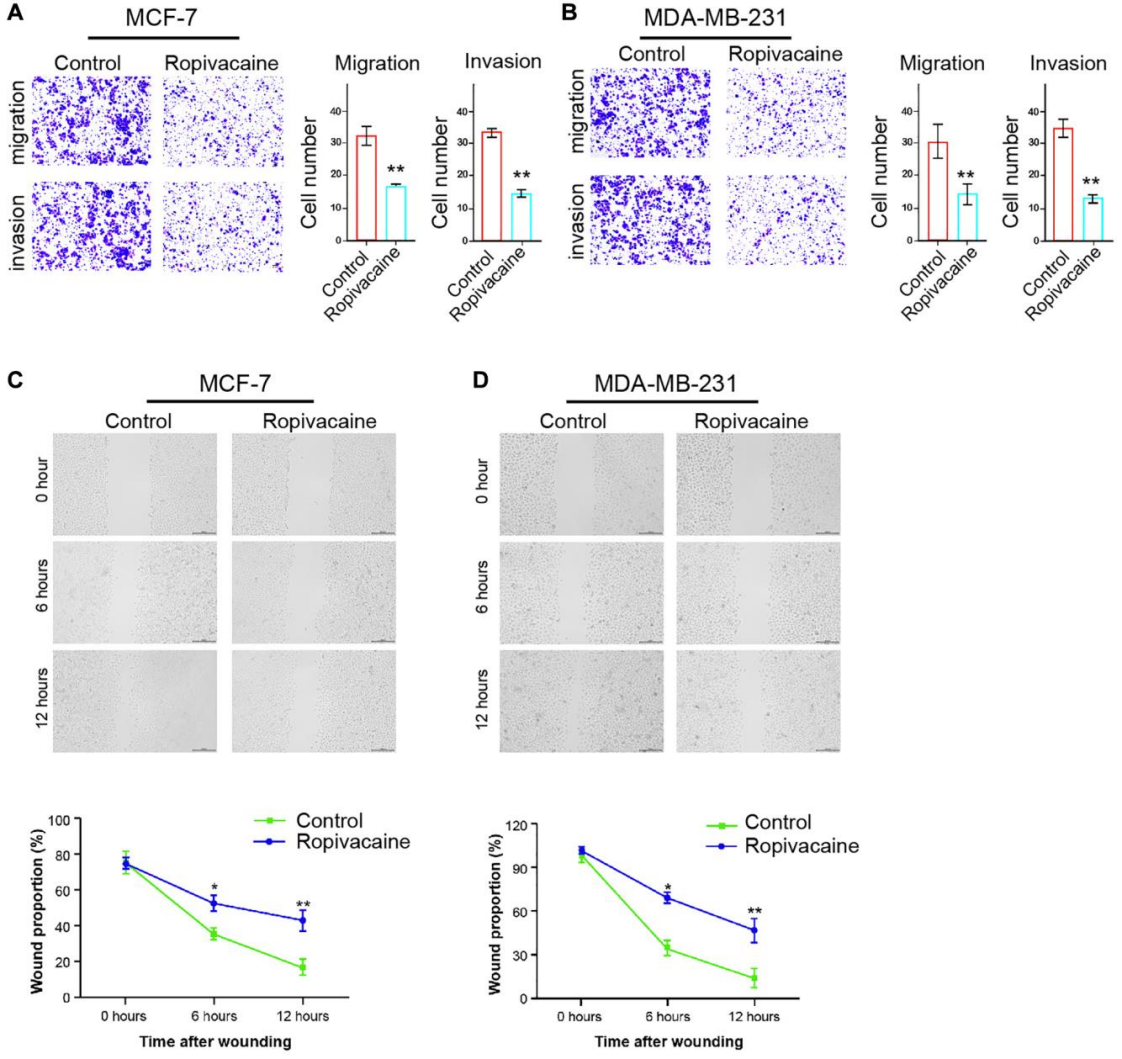
**Ropivacaine decreases invasion and migration of breast cancer cells.** (**A**–**D**) The MCF-7 and MDA-MB-231 cells were treated with ropivacaine (1 mmol/L) or equal volume saline. (**A** and **B**) The cell migration and invasion were examined by transwell assays in the cells. (**C** and **D**) The migration and invasion were measured by wound healing assays in the cells. The wound healing proportion was shown. *N* = 3, The independent experiments were repeated for three times. Data are presented as mean ± SD. Statistic significant differences were indicated: ^*^P < 0.05, ^**^P < 0.01.

We then detected the influence of ropivacaine on regulating breast cancer cell invasion and migration. The invasion and migration of MCF-7 and MDA-MB-231 cells were significantly reduced by the treatment of ropivacaine (Figure 2A and 2B). Ropivacaine notably increased proportion of wound healing in MCF-7 and MDA-MB-231cells (Figure 2C and 2D).

### Ropivacaine enhances the expression of miR-27b-3p in breast cancer cells

**Figure 3:**
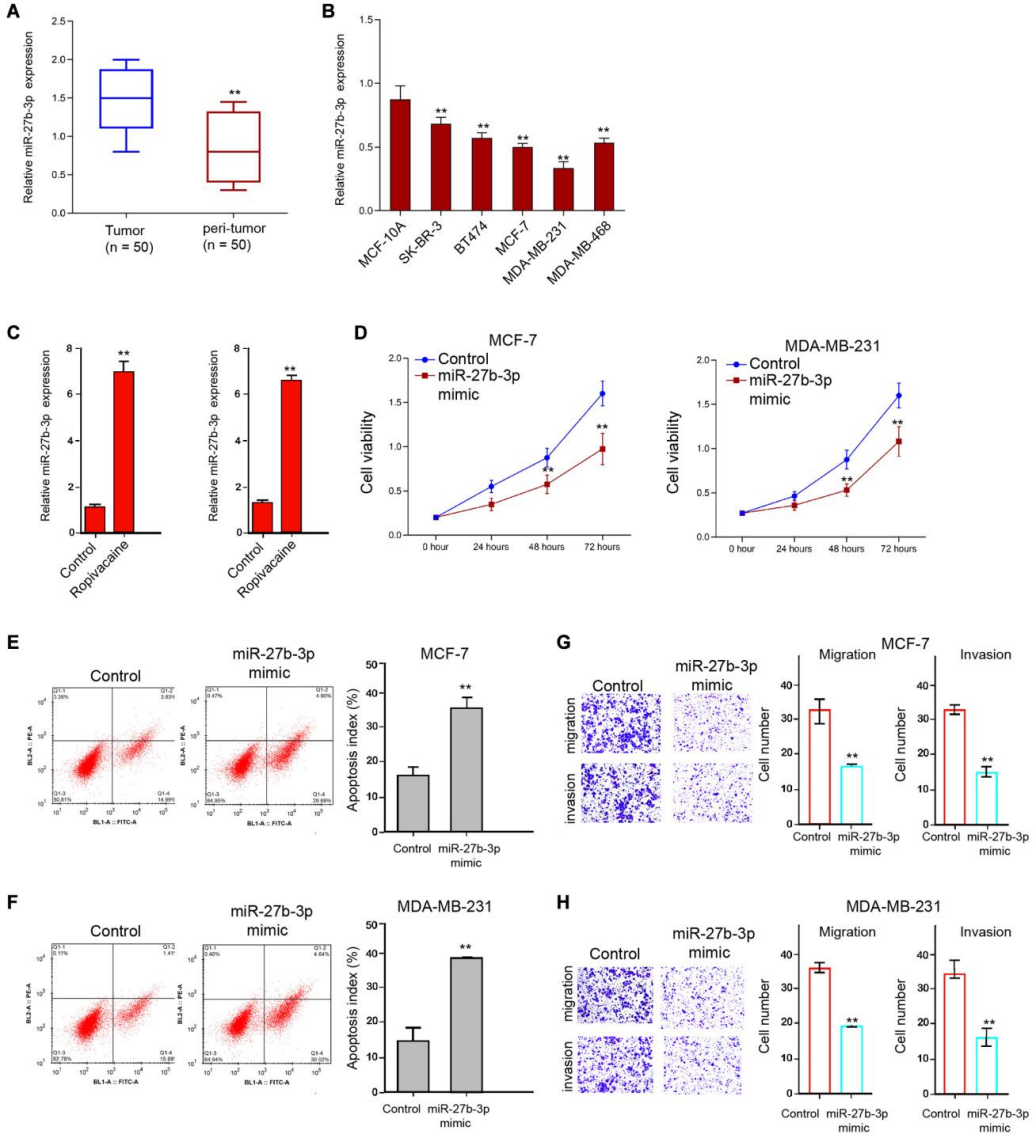
**Ropivacaine enhances the expression of miR-27b-3p in breast cancer cells.** (**A**) The expression of miR-27b-3p was detected in the tumor and peri-tumor tissues from breast cancer patients (*n* = 50). (**B**) The expression of miR-27b-3p was analyzed in the indicated cells. (**C**) The MCF-7 and MDA-MB-231 cells were treated with ropivacaine (1 mmol/L) or equal volume saline. The expression of miR-27b-3p was measured by qPCR assays in the cells. (**D**–**H**) The MCF-7 and MDA-MB-231 cells were treated with control mimic or miR-27b-3p mimic (50 nM) for 48 hours. (**D**) The cell viability was analyzed by the MTT assays in the cells. (**E** and **F**) The cell apoptosis was measure by flow cytometry analysis in the cells. (**G** and **H**) The cell migration and invasion were examined by transwell assays in the cells. *N* = 3, The independent experiments were repeated for three times. Data are presented as mean ± SD. Statistic significant differences were indicated: ^*^P < 0.05, ^**^P < 0.01.

Then, we explored the mechanisms of ropivacaine-exerted inhibition on breast cancer. MiR-27b-3p was reduced in tumor tissues compared with that in peri-tumor tissues from breast cancer patients (*n* = 50) (Figure 3A). Meanwhile, we analyzed miR-27b-3p expression in MCF-10A, Her2+ SK-BR-3 cell lines, luminal BT474 and MCF-7 cell lines, and TNBC MDA-MB-231 and MDA-MB-468 cells lines, and miR-27b-3p presented the lowest expression, which was used in the subsequent analysis (Figure 3B). We identified that ropivacaine was able to up-regulate the expression of miR-27b-3p (Figure 3C). Moreover, the treatment of miR-27b-3p mimic was able to inhibit the cell viability (Figure 3D). Meanwhile, cell apoptosis was increased by the treatment of miR-27b-3p mimic in the cells (Figure 3E and 3F). In addition, cell invasion and migration were repressed by miR-27b-3p mimic (Figure 3G and 3H). Together these data suggest that ropivacaine may induce the inhibitory effect on breast cancer progression by up-regulating miR-27b-3p.

### MiR-27b-3p targets YAP in breast cancer cells

**Figure 4:**
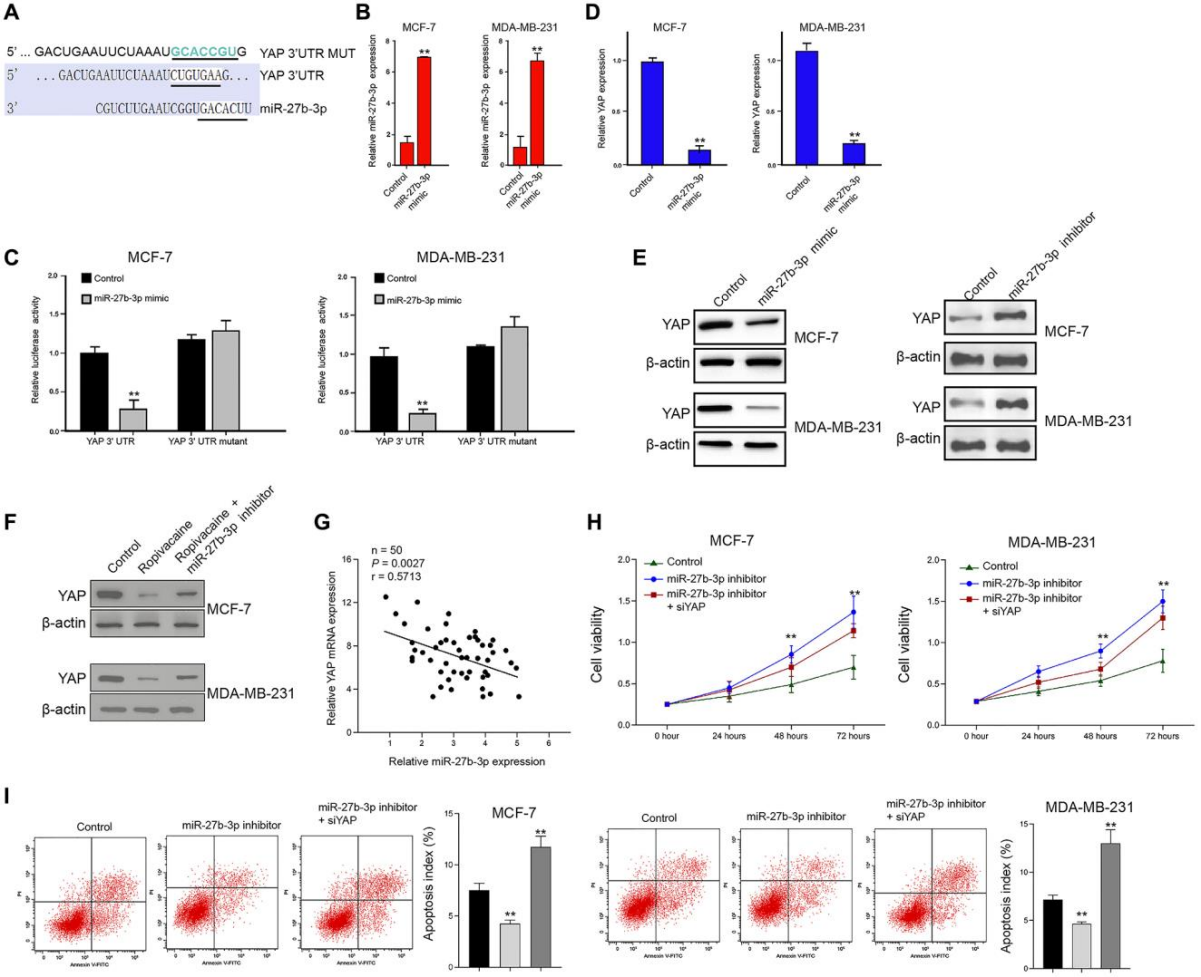
**MiR-27b-3p targets YAP in breast cancer cells.** (**A**) The interaction of miR-27b-3p and YAP 3’ UTR was identified by bioinformatic analysis using Targetscan (http://www.targetscan.org/vert_72/). (**B**–**D**) The MCF-7 and MDA-MB-231 cells were treated with control mimic or miR-27b-3p mimic. (**B**) The expression of miR-27b-3p was tested by qPCR assays in the cells. (**C**) The luciferase activities of wild type YAP 3’ UTR (YAP WT) and YAP 3’ UTR with the miR-27b-3p-binding site mutant (YAP MUT) were determined by luciferase reporter gene assays in the cells. (**D**) The mRNA expression of YAP was measured by qPCR assays in the cells. (**E**) The MCF-7 and MDA-MB-231 cells were treated with miR-27b-3p inhibitor or miR-27b-3p mimic. The protein expression of YAP was analyzed by Western blot analysis in the cells. (**F**) The MCF-7 and MDA-MB-231 cells were treated with ropivacaine (1 mmol/L) or equal volume saline, or co-treated with ropivacaine (1 mmol/L) and miR-27b-3p inhibitor. The protein expression of YAP was measured by Western blot analysis in the cells. (**G**) The expression of miR-27b-3p and YAP was detected in the tumor tissues from breast cancer patients (*n* = 50). (**H**) The cell viability was analyzed by MTT assays in the indicated cells. (**I**) The cell apoptosis was measure by flow cytometry analysis in the indicated cells. *N* = 3, The independent experiments were repeated for three times. Data are presented as mean ± SD. Statistic significant differences were indicated: ^**^P < 0.01.

Then, we identified the miR-27b-3p-targeted site in YAP 3′ UTR by using Targetscan (http://www.targetscan.org/vert_72/) (Figure 4A). To determine the effect of miR-27b-3p on YAP, the MCF-7 and MDA-MB-231 cells were treated with miR-27b-3p mimic, and the efficiency was validated in the cells (Figure 4B). Notably, the miR-27b-3p mimic treatment inhibited the luciferase activities of wild type YAP 3′ UTR but failed to affect the YAP 3′ UTR with the miR-27b-3p-binding site mutant in the cells (Figure 4C). Furthermore, the mRNA and protein expression of YAP were significantly reduced by miR-27b-3p mimic but were enhanced by miR-27b-3p inhibitor in the cells (Figure 4D and 4E), suggesting that miR-27b-3p is able to target YAP. Moreover, the expression of YAP was inhibited by the treatment of ropivacaine, in which the miR-27b-3p inhibitor could rescue this effect in the cells (Figure 4F). The expression of miR-27b-3p was negatively correlated with YAP expression in tumor tissues from breast cancer patients (Figure 4G). Moreover, the miR-27b-3p inhibitor enhanced proliferation and reduced apoptosis of breast cancer cells, which were reversed by YAP knockdown using siRNA Figure 4H and 4I).

### Ropivacaine inhibits the progression of breast cancer by miR-27b-3p /YAP axis *in vitro*

**Figure 5:**
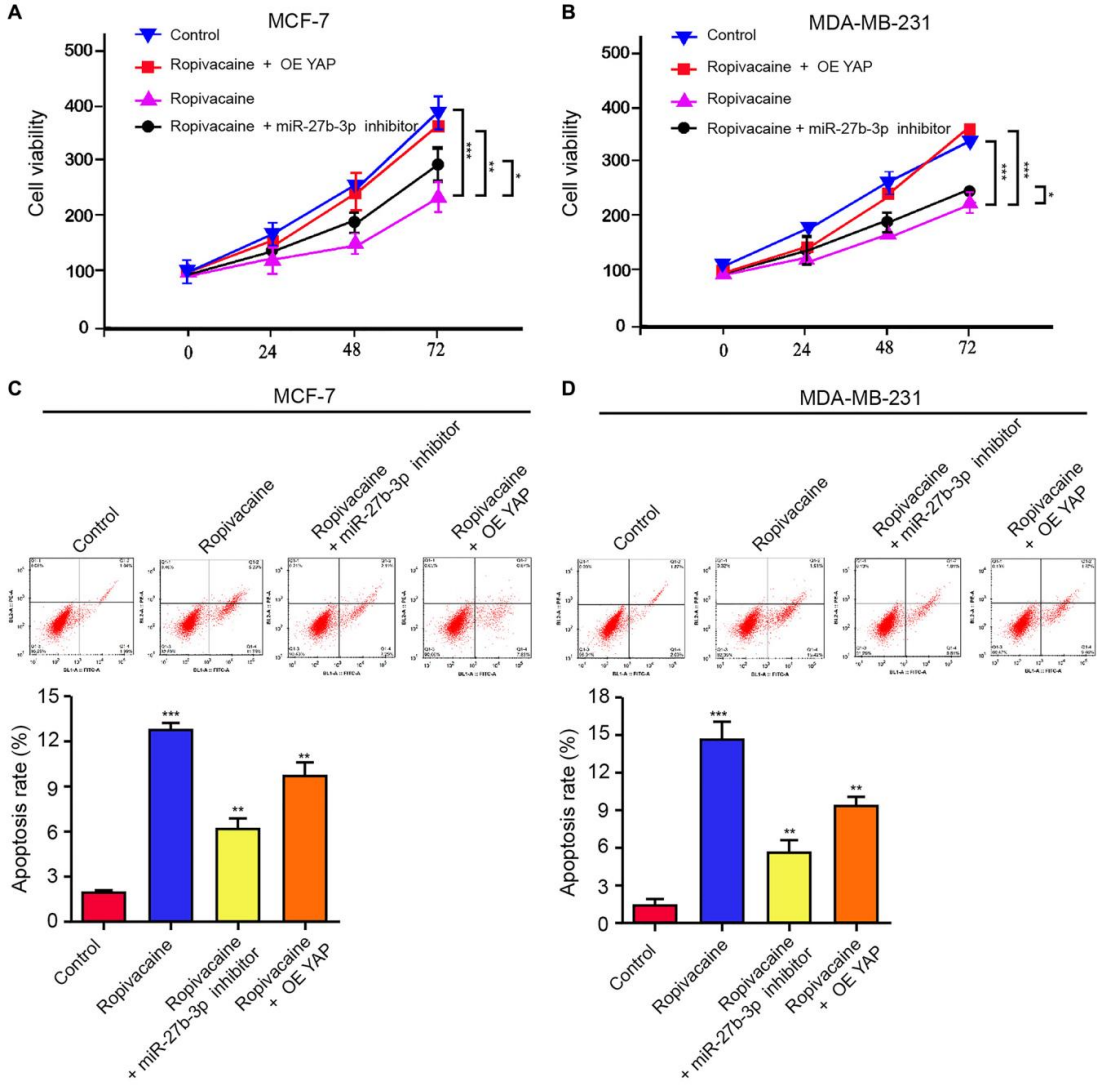
**Ropivacaine inhibits the progression of breast cancer by miR-27b-3p /YAP axis *in vitro*.** (**A**–**D**) The MCF-7 and MDA-MB-231 cells were treated with ropivacaine (1 mmol/L) or equal volume saline, or co-treated with ropivacaine (1 mmol/L) and miR-27b-3p inhibitor or YAP overexpression vector. (**A** and **B**) The cell viability was analyzed by the MTT assays in the cells. (**C** and **D**) The cell apoptosis was measure by flow cytometry analysis in the cells. *N* = 3, The independent experiments were repeated for three times. Data are presented as mean ± SD. Statistic significant differences were indicated: ^*^P < 0.05, ^**^P < 0.01, ^***^P < 0.001.

We then determined the role of ropivacaine/miR-27b-3p/YAP axis in breast cancer pathogenesis. Ropivacaine inhibited cell viabilities of MCF-7 and MDA-MB-231 cells, while miR-27b-3p inhibitor or YAP overexpression rescued this inhibition (Figure 5A and 5B). The miR-27b-3p inhibitor or the overexpression of YAP repressed the apoptosis induced by ropivacaine in MCF-7 and MDA-MB-231cells (Figure 5C and 5D). Together these indicate that ropivacaine inhibits the progression of breast cancer by modulating miR-27b-3p/YAP axis *in vitro.*

### Ropivacaine attenuates the cell growth of breast cancer *in vivo*

**Figure 6:**
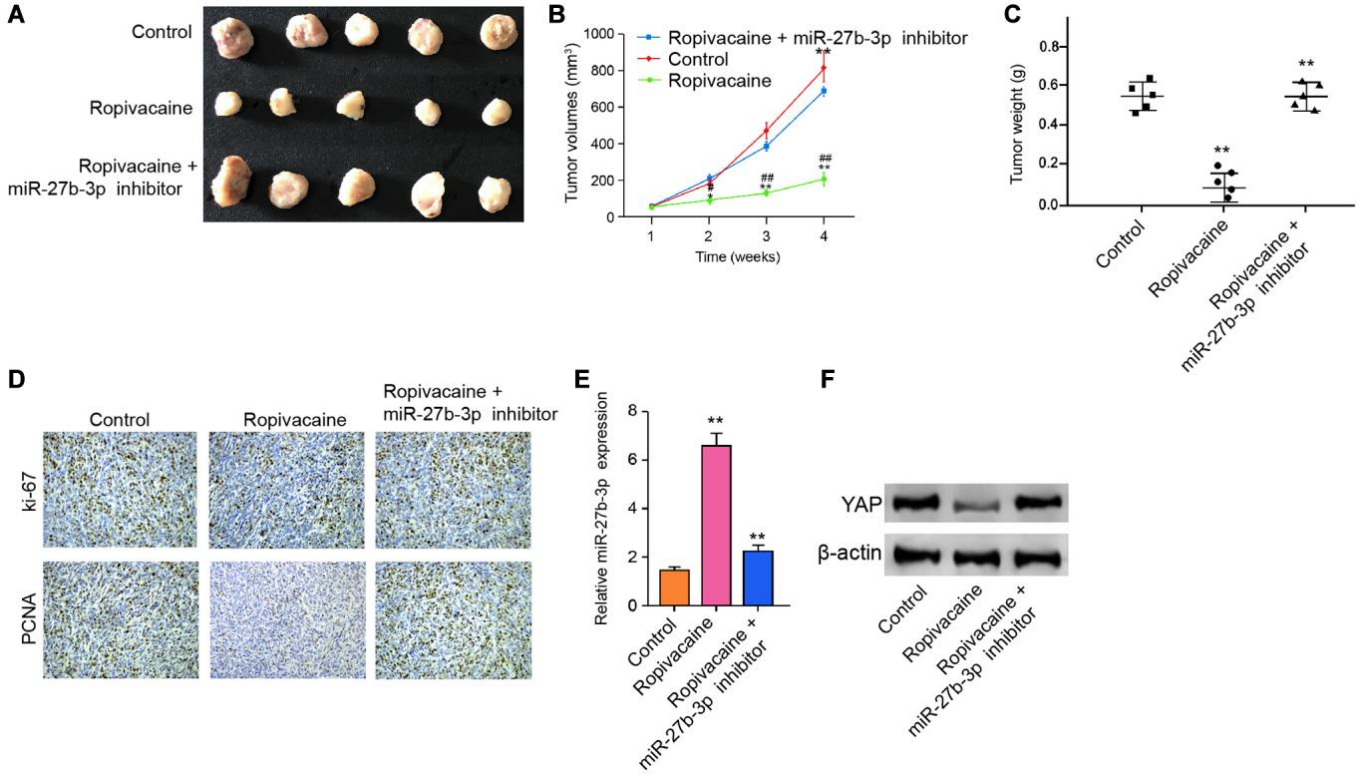
**Ropivacaine attenuates the tumor growth of breast cancer *in vivo*.** (**A**–**F**) The effect of ropivacaine on tumor growth of breast cancer cells *in vivo* was analyzed by nude mice tumorigenicity assay by injected with the MDA-MB-231 cells. The mice (*n* = 5) were treated with equal volume saline, or ropivacaine (40 μmol/Kg), or co-treated with ropivacaine (40 μmol/Kg) and miR-27b-3p inhibitor. (**A**) Representative images of dissected tumors from nude mice were presented. (**B**) The average tumor volume was calculated and shown. (**C**) The average tumor weight was calculated and shown. (**D**) The levels of ki-67 and PCNA were measured by immunohistochemistry analysis in the mice. (**E**) The expression levels of miR-27b-3p were examined by qPCR assays in the tumor tissues of the mice. (**F**) The expression levels of YAP were measured by qPCR assays in the tumor tissues of the mice. Data are presented as mean ± SD. Statistic significant differences were indicated: ^##^*P* < 0.0, ^**^P < 0.01.

We then assessed the function of ropivacaine in breast cancer cell growth *in vivo*. We performed the tumorigenicity assays in nude mice by injecting with MDA-MB-231 cells and treating with ropivacaine or co-treating with ropivacaine and miR-27b-3p inhibitor. The treatment of ropivacaine repressed the cell growth of MDA-MB-231 cells *in vivo*, while miR-27b-3p inhibitor could reverse this effect, as shown by tumor size (Figure 6A), tumor volume (Figure 6B), tumor weight (Figure 6C), and the levels of ki-67 and PCNA (Figure 6D). Besides, the expression of miR-27b-3p was increased by ropivacaine in the tumor tissues of the mice (Figure 6E). The treatment of ropivacaine down-regulated the expression of YAP in the tumor tissues of the mice, in which miR-27b-3p inhibitor could rescue this phenotype (Figure 6F). These results suggest that ropivacaine inhibits the cell growth of breast cancer *in vivo.*

## DISCUSSION

Breast cancer is a prevalent cancer with high mortality as well as poor prognosis [22]. As a broadly utilized anesthetic, ropivacaine shows anti-tumor activities. Ropivacaine represses cervical cancer cells proliferation by suppressing the miR-96/MEG2/pSTAT3 signaling [23]. Ropivacaine restrains cancer angiogenesis through the dysfunction of sodium-channel-independent mitochondria and oxidative stress [24]. Ropivacaine inhibits gastric cancer development by down-regulating the phosphorylation of ERK1/2 [25]. The impact of ropivacaine on metastatic and primary colon cancer cells has been reported [11]. Ropivacaine hinders migration, invasion, and proliferation and increases apoptosis of papillary thyroid cancer cells via ITGA2 [26]. Ropivacaine represses the invasion, migration, and growth of gastric cancer by attenuating PI3K/AKT and WEE1 signaling through miR-520a-3p [27]. Our results showed that ropivacaine repressed breast cancer cell malignant phenotypes. Ropivacaine suppressed cell growth of breast cancer *in vivo*. It presents an innovative anti-tumor effect of ropivacaine on breast cancer, indicating interesting data for the important function of anesthetic in modulating breast cancer. The concentration of the anti-breast cancer application of ropivacaine in the clinic is an unresolved challenge and the drug combination strategy of ropivacaine with other anti-cancer treatments may partly relieve this problem, which needs to further explore in the future.

It has been identified that microRNA-143 is able to target MAPK3 to control bone metastasis and proliferation in breast cancer cells [28]. MiR-589-3p increases apoptosis and represses the invasion, migration, and proliferation of breast cancer cells by overcoming the Akt signaling through IGF1R [29]. MiR-1976 depletion elevates cancer stem cell properties and epithelial-mesenchymal transition to induce breast cancer metastasis [30]. MicroRNA-4500 restrains angiogenesis, invasion, and migration of breast cancer cells [31]. Moreover, it has been reported that miR-27b-3p represses proliferation and converts multi-chemoresistance [32]. The down-regulation of miR-27b-3p promotes tamoxifen resistance in breast cancer [33]. Our mechanical investigation further demonstrated that ropivacaine could enhance the expression of miR-27b-3p in breast cancer cells and miR-27b-3p inhibited breast cancer progression *in vitro*. It displays a critical relationship between ropivacaine and miR-27b-3p in breast cancer, presenting the mechanism of ropivacaine-regulated anti-cancer effect.

It has been revealed that TNFα/YAP/p65/HK2 signaling is able to mediate the migration of breast cancer cells [34]. YAP increases the metastasis of breast cancer by inhibiting growth differentiation factor-15 [35]. MiR-520b enhances the stemness of breast cancer by modulating Hippo/YAP signaling [36]. RUNX1 and RUNX3 preserve against YAP-induced shorter survival outcomes, stemness, and EMT in breast cancer [37]. YAP/TAZ-induced serine metabolism activation and methylation modulation are crucial for LKB1-knockdown breast cancer development [38]. Meanwhile, miR-27b-3p reduces chemoresistance and progression of breast cancer [32]. MiR-27b-3p attenuates tamoxifen resistance of breast cancer cells [33]. Our data showed that YAP was targeted by miR-27b-3p and involved in ropivacaine/miR-27b-3p-suppressed breast cancer progression. These data provide new evidence that ropivacaine regulates YAP through miR-589-3p in breast cancer development. It has been identified that miR-27a-3p works on YAP in oral squamous cell carcinoma cells [39]. The bioinformatics analysis identified the potential binding sites of miR-27a-3p and miR-27b-3p within YAP mRNA 3′ UTR, and we validated that miR-27a-3p failed to regulate YAP expression in breast cancer cells (data not shown). MiR-27b-3p inhibits breast cancer proliferation but miR-27a-3p enhances breast cancer progression [32, 40], highlighting the therapeutic importance of miR-27b-3p in breast cancer. Our data indicated that ropivacaine/miR-27b-3p represses YAP expression by at least partially targeting its mRNA 3′ UTR. Ropivacaine/miR-27b-3p may affect YAP expression by other mechanisms and is needed to further explore. Meanwhile, the overexpression of YAP and the miR-27b-3p inhibitor did not fully rescue ropivacaine-reduced breast cancer cell viability. It suggests that ropivacaine may induce the inhibitory effect on breast cancer progression by other mechanisms as well, which need to investigate in the future.

In summary, we concluded that the anesthetic ropivacaine attenuated the breast cancer *via* the miR-27b-3p/YAP axis. Ropivacaine may be utilized as anti-cancer agent in breast cancer.

## MATERIALS AND METHODS

### Cell culture

Human mammary epithelial cell line MCF-10A, Her2+ SK-BR-3 cell lines, luminal BT474 and MCF-7 cell lines, and TNBC MDA-MB-231 and MDA-MB-468 cells lines were obtained in American Type Tissue Culture Collection. Cells were incubated at a condition of 37°C with 5% CO_2_ in DMEM (Gibco, USA) with 100 units/mL penicillin (Gibco, USA), 0.1 mg/mL streptomycin (Gibco, USA), and fetal bovine serum (10%, Gibco, USA). Ropivacaine (Sigma, USA) was dissolved by DMSO and diluted in PBS. The miR-27b-3p mimic and inhibitor were purchased (GenePharma, China). The pcDNA3.1-YAP overexpression vectors and YAP siRNA were obtained using full-length synthesis (GenePharma, China). Liposome 3000 (Invitrogen, USA) was utilized for the transfection.

### Tissue samples of patients

The tumor and peritumor tissues of breast cancer patients (*n* = 50) were obtained underwent surgical resections from Handan Central Hospital under the informed consent. The experiments conformed with authorization of Ethics Committee of Handan Central Hospital.

### Quantitative reverse transcription-PCR (qRT-PCR)

The TRIZOL (Invitrogen, USA) was applied to extract RNAs from the cells and tissues, and the total RNAs were subjected into cDNA synthesis (Thermo, USA). The SYBR Real-time PCR I kit (Takara, Japan) was applied to the qRT-PCR. The primer sequences are as follows: GAPDH, sense, 5′-CTTTGGTATCGTGGAAGGACTC-3′; anti-sense, 5′-GTAGAGGCAGGGATGATGTTCT-3′; U6, sense, 5′-GCTTCGGCAGCACATATACTAAAAT-3′; anti-sense, 5′-CGCTTCACGAATTTGCGTGTCAT-3′; miR-27b-3p sense: 5′- CGCCTTGAATCGGTG-3′; miR-27b-3p anti-sense: 5′- GTGCAGGGTCCGAGGT-3′; YAP sense: 5′-AGGAGAGACTGCGGTTGAAA-3′; YAP anti-sense: 5′-CCCAGGAGAAGACACTGCAT-3′.

### MTT

Cells were treated as indicated in each experiment, digested, suspended as single cells, and planted in 96-well plates (2 × 10^4^ cells per well). At the end time point, MTT reagent were added at a final concentration of 5 mg/ml in each well. Following a 4-hours incubation, the cell medium was discarded and replaced by 150 μl DMSO in each well. The plates were gently shaken in dark for 10 minutes. Absorbance at 570 nm was detected.

### Colony formation assays

Cells were digested and suspended in DMEM as single cell. A total number of 1000 cells were seeded into each well of 6-well plate. The cells were cultured for 14 days until visible clones formed. Subsequently, the clones were fixed, stained by violate crystal (Sigma) for 30 minutes at room temperature and captured in a microscope (Olympus, Japan).

### Transwell assays

The transwell chambers (Corning, USA) covered with or without Matrigel (Corning) were used to check the invasion or migration ability of MDA-MB-231 and MCF-7 cells. Cells were placed in the upper chamber with DMEM medium containing no FBS, while the lower chambers were filled with complete culturing medium with 10% FBS. After incubation for 48 hours, the upper chambers were washed with PBS and stained with violate crystal.

### Wound healing assay

Wound healing assay was performed to determine cell migration. MDA-MB-231 and MCF-7 cells were placed in 6-well plates at a density of 3 × 10^5^ per well and cultured for 12 hours to form a monolayer confluence. Then a 200 μL pipet was used to gently scratch a line on the monolayer. Then the medium was replaced by fresh FBS-free medium. The pictures of scratches were taken at 0 h, 6 h and 12 h.

### Analysis of cell apoptosis

The portion of apoptotic cells after indicated transfection were detected by flow cytometry. Cells were seeded in 6-well plates and subjected to indicated treatment, collected and stained with PI and FITC-Annexin V (CST, USA) for 15 minutes, then the fluorescence density was examined by a Flow cytometry (BD Biosciences) immediately.

### Luciferase reporter gene assay

The potential binding sites between miR-27b-3p with 3′ UTR region of YAP were analyzed. The wild type sequence of YAP was downloaded from PubMed website, cloned, and inserted into the pmirGLO plasmid (GenScript, China). pmirGLO-YAP were co-transfected with control mimic or miR-27b-3p mimic into cells. After transfection for 24 hours, the luciferase activity was examined by a dual luciferase reporter assay kit under the manufacturer’s instruction (Promega, USA).

### Western blot analysis

Tissues and cells and were harvested and lysed by RIPA (CST, USA) added with 1× protease inhibitor cocktail (Beyotime). Protein concentration was evaluated by a BCA quantification method. Subsequently, 35 μg extracted samples were separated on SDS-PAGE and transferred onto PVDF membranes. The blot was probed using specific primary antibodies (YAP (Abcam, USA), and β-actin (Abcam, USA)) at 4°C overnight, followed by incubation with secondary antibodies at room temperature for 1 hour. The blots were washed with TBST for three times, followed by visualization via a Millipore ECL reagent (Millipore, USA) under a Gel imaging system (BD Biosciences, USA).

### Tumorigenicity analysis

The influence of ropivacaine on cell growth of breast cancer was detected in male and 4-week-old Balb/c nude mice (*n* = 5) by subcutaneously injected with 1 × 10^7^ cells MDA-MB-231 cells. After 7 days, we measured tumor size every 1 week. Tumors were scaled after 4 weeks of injection. The tumor size was calculated via the following formula: length × width2/2. Levels of ki-67 and PCNA were detected by immunohistochemistry analysis in the mice. Methods procedures were approved by Animal Ethics Committee of Handan Central Hospital.

### Immunohistochemistry analysis

The fixed tissues were subjected to immunohistochemistry analysis. The section was deparaffinized with xylene, dehydrated in ethanol in a concentration gradient and was subjected into quench endogenous peroxidase. Citrate buffer (10 mM, pH 6.0) were utilized to antigen retrieval about 10 minutes in a microwave. Nonspecific-binding site was blocked using normal goat serum for 30 minutes. The paraffin slices were incubated with the primary antibody at 4°C overnight. The anti-mouse IgG was plated into the section for 30 minutes. DAB substrate was applied for color presentation.

### Statistical analysis

The data analysis was performed *via* GraphPad prism 7. Data were shown as mean ± SD. An unpaired Student’s *t*-tests were applied for comparison between two experimental groups, while the one-way ANOVA method was applied to compare the difference between multiple groups. *P* < 0.05 were set as the threshold for statistical significance.
